# Supplementary material for: Spin Exchange Interaction in Substituted Copper Phthalocyanine Crystalline Thin Films
Source: Sci Rep. 2015 Nov 12;5:16536. doi: 10.1038/srep16536 (PMC4642266; doi:10.1038/srep16536)
Supplement: Supplementary Information [file srep16536-s1.pdf]

Supplementary Materials
Spin Exchange Interaction in Substituted Copper Phthalocyanine Crystalline Thin Films

Naveen Rawat,[1,*] Zhenwen Pan,[1] Cody J. Lamarche,[1] Anthony Wetherby,[2] Rory Waterman,[2] Takahisa Tokumoto,[3] Judy G. Cherian,[3] Randall L. Headrick,[1] Stephen A. McGill,[3] and Madalina I. Furis[1,†]

[1] *Department of Physics and the Material Science Program, University of Vermont, Burlington, Vermont 05405, USA*
[2] *Department of Chemistry, University of Vermont, Burlington, Vermont 05405, USA*
[3] *National High Magnetic Field Laboratory, Tallahassee, FL 32310, USA*

[*] Naveen.Rawat@uvm.edu

[†] Madalina.Furis@uvm.edu

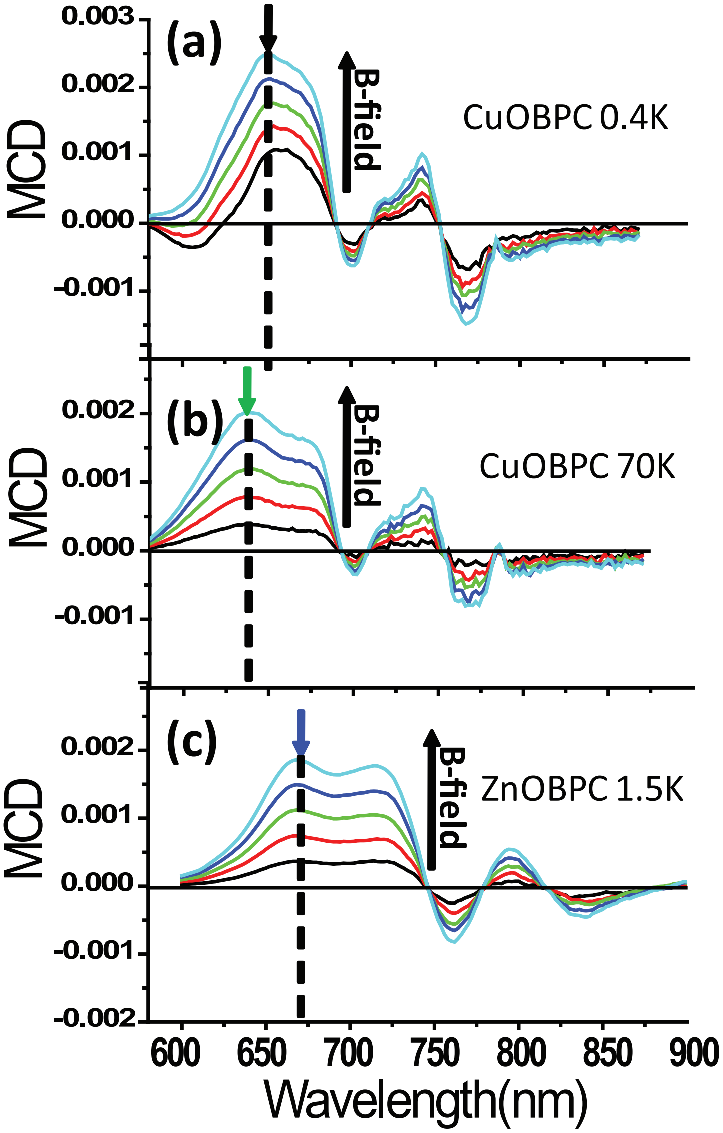

Supplementary Figure S 1. MCD spectra measured for (a) CuOBPC at 0.4K, (b) CuOBPC at 70K, and (c) ZnOBPC at 1.5K. Spectra are recorded from 2 T to 10 T in 2 T increments. ZnOBPC is a closed shell system with no magnetic exchange between Zn ions and only diamagnetic behavior is observed. The derivative like shape observed on the high energy side of the low temperature Cu-OBPc spectrum evolves into a Gaussian-like shape associated with a non degenerate state at high temperatures.

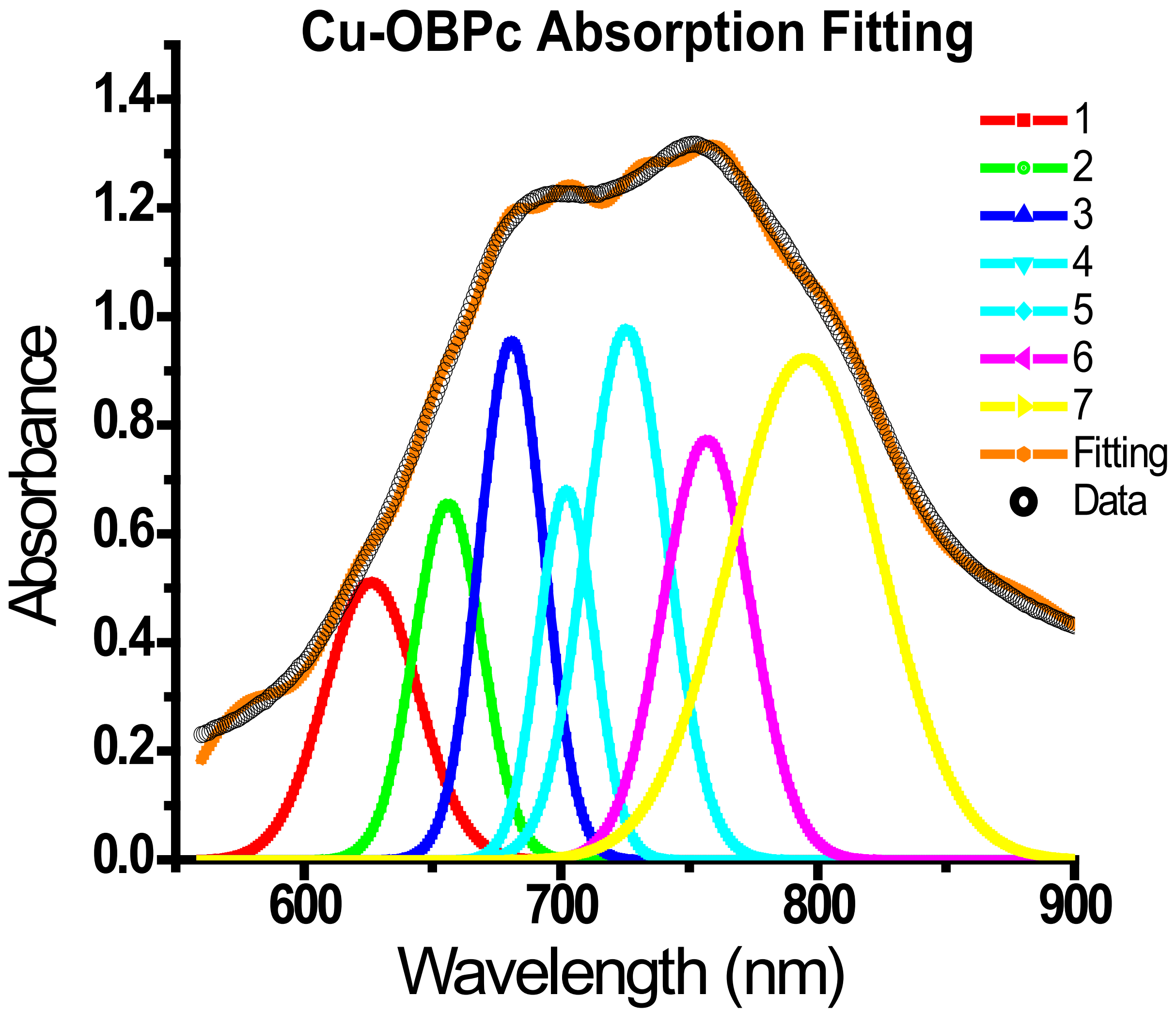

Supplementary Figure S 2. (a) Absorption fitting of Cu-OBPc polycrystalline thin film
